# Hypokalemic Paralysis Secondary to Immune Checkpoint Inhibitor Therapy

**DOI:** 10.1155/2017/5063405

**Published:** 2017-11-08

**Authors:** Pragathi Balakrishna, Augusto Villegas

**Affiliations:** ^1^PGY3 Internal Medicine, Orange Park Medical Center, Orange Park, FL, USA; ^2^Department of Hematology and Oncology, Orange Park Medical Center, Orange Park, FL, USA

## Abstract

Introduction of immune checkpoint inhibitors (ICIs) has led to significant improvements in the treatment of multiple malignancies. Anti-programmed cell death protein 1 (PD-1) and anti-cytotoxic T-lymphocyte antigen 4 (CTLA-4) are two essential ICIs that have been FDA approved since 2011. As the use of immunotherapy in melanoma and other malignancies increases, the potential of adverse events also increases. Overall, anti-PD-1 agents are well tolerated. In rare instances, colitis, endocrinopathies, skin, and renal toxicities have been observed. A 58-year-old male with a history of stage 4 cutaneous melanoma presented with quadriplegia while on nivolumab. Routine blood test revealed low potassium, low bicarbonate, and high serum creatinine. Admission diagnosis included hypokalemia, acute kidney injury, and renal tubal acidosis. The offending drug was discontinued, and the patient was started on high-dose corticosteroids. On discharge, paralysis was resolved. Renal function and potassium were normalized. Nivolumab was discontinued, and he was started on pembrolizumab. Literature suggests that, although rare, patients receiving ICE may develop immune-mediated nephritis and renal dysfunction. The mainstay of immune-related adverse event (irAE) management is immune suppression. Hence, given the increasing frequency of immunotherapy use, awareness should be raised in regard to irAEs and their appropriate management.

## 1. Introduction

**Figure 1:**
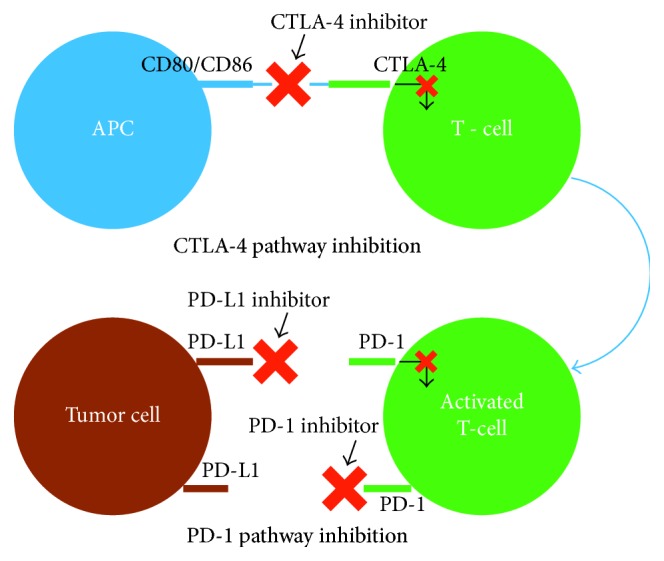
Mechanism of action of immune checkpoint inhibitors. CTLA-4 = cytotoxic T-lymphocyte antigen 4, PD-1 = programmed cell death protein 1, PD-L1 = programmed death-ligand 1.

Immune checkpoint inhibitors (ICIs) are emerging as revolutionary drugs targeting a variety of malignancies such as melanoma, non-small-cell lung carcinoma (NSCLC), and renal cell carcinoma (RCC) [1]. Three checkpoint protein inhibitory antibodies have been FDA approved for melanoma since 2011. These are nivolumab and pembrolizumab, which block programmed death 1 receptor (PD-1), and ipilimumab, which blocks cytotoxic T-lymphocyte-associated antigen 4 (CTLA-4) receptor [2]. PD-1 transmits inhibitory signals to immune cells, leading to decreased proliferation and apoptosis. Cancer cells express PD-L1, the ligand of PD-1, allowing the tumor to escape attack by effector T cells. Immune checkpoint inhibitors encompass blocking antibodies to the PD-1/PD-L1 and CTLA-4 checkpoint molecules. These drugs block the PD-1/PD-L1 interaction, enhancing the cellular response against the tumor (Figure 1) [3].

Nivolumab and pembrolizumab are anti-PD-1 antibodies which are indicated for metastatic melanoma. They have shown superior progression-free survival (PFS) among patients with advanced melanoma [4, 5]. A trial comparing nivolumab to dacarbazine in previously untreated patients showed a 12-month survival rate of 73% versus 42%, respectively [6]. Another phase 1 clinical trial studied the progression-free survival amongst patients with advanced melanoma treated with pembrolizumab. This trial showed a 12-month progression-free survival rate of 35% in patients regardless of previous ipilimumab treatment and a 12-month progression-free survival rate of 52% in treatment-naïve patients [5].

On the one hand, ICIs have dramatically improved the outcome of metastatic melanoma and other cancers such as NCSLC and RCC [7]. However, the therapy is associated with immune-related adverse events (irAEs), which were observed in 5% of patients [4]. The basis for the majority of these adverse events is a hyperactivated T-cell response with reactivity directed against normal tissue, resulting in the generation of high levels of CD4 T-helper cell cytokines or increased migration of cytolytic CD8 T cells within normal tissues [2]. Overall, anti-PD-1 agents are well tolerated with low-grade fatigue, diarrhea, pruritus, nausea, and decreased appetite occurring as the most common adverse events. Sometimes colitis, endocrinopathies, skin toxicity, and renal toxicities have been observed [8]. Acute renal failure has been reported in < 1% of the patients treated with nivolumab monotherapy or in combination studies for melanoma or NSCLC. Patients with renal injury typically present with elevations in serum creatinine and are treated with steroids which lead to clinical improvement and resolution in most cases [9]. Here, we present a patient with advanced melanoma who developed metabolic acidosis, hypokalemic paralysis, and acute renal failure during treatment with anti-PD1 antibodies.

## 2. Case Report

A 58-year-old Caucasian male with a history of metastatic melanoma presented with severe weakness. The patient was initially diagnosed with cutaneous melanoma of the back in May 2008 (American Joint Committee of Cancer (AJCC) Stage 1b; T2a, N0, M0; BRAF V600 mutation not detected) and underwent wide local excision with clear margins. In April 2014, he presented with progressive shortness of breath and significant weight loss over the past 3–4 weeks. CT angiography of the chest showed mediastinal and hilar lymphadenopathy, along with numerous pulmonary nodules throughout both lungs ranging between 6 mm and 3.5 cm. CT abdomen and MRI brain were done which ruled out any liver lesion or brain metastasis. Biopsy of the lung nodules confirmed metastatic melanoma (BRAF V600 mutation not detected), and treatment with anti-CTLA-4 antibody, ipilimumab (3 mg/kg), was initiated. The patient received this treatment regimen for 3 months but then was lost to follow-up. In November 2014, he agreed to start therapy with nivolumab, an anti-PD- L1 antibody. Ten months later, he presented with progressive quadriplegia, lower extremity weakness worse than upper extremity weakness, developing over a few days. He reported that, due to the weakness, he fell and was unable to get up on his own. His home medication list included only nivolumab. Routine blood test in ED revealed potassium of 1.7 mEq/L, bicarbonate of 9 mEq/L, chloride of 116 mEq/L, sodium of 139 mEq/L (anion gap 14, delta ratio of 2), serum creatinine of 2.64 mg/dL (baseline of 0.91 mg/dL 3 weeks prior), and elevated eosinophils of 6%. Admission diagnosis included hypokalemia, hyperchloremic normal anion gap metabolic acidosis, acute kidney injury (AKI), and eosinophilia. Urinalysis revealed 1+ protein (urine protein/creatinine ratio of 1.42), fractional excretion of sodium of 1.9%, amorphous urine sediment, and 24-hour urine potassium of 159 mEq/L, hence indicating intrinsic kidney injury such as acute interstitial nephritis, acute tubular necrosis, or glomerulonephritis. Pertinent negatives in his blood work included normal thyroid-stimulating hormone levels. Retroperitoneal ultrasound showed normal kidneys with no solid mass, stone, or obstruction. Urinary bladder also appeared normal. There was no history suggestive of previous kidney disease; autoimmune diseases such as sjögrens, lupus nephritis, or rheumatoid arthritis (RA); thyroid disorders; diabetes; vomiting; excessive alcohol use; dehydration; or exposure to nephrotoxic agents such as antibiotics, contrast, or analgesics. Based on these findings, progressive quadriplegia was secondary to hypokalemia which was due to type 1 RTA, most likely irAE secondary to anti-PD-1 antibody therapy. The offending drug was discontinued, and the patient was started on intravenous hydrocortisone 100 mg every 6 hours for 2 days, followed by a tapering dose lasting 4 weeks. IV replacement therapy with potassium and bicarbonate was also initiated. Renal biopsy was considered but was not done as the patient responded well to steroids with return of renal function to normal. Quadriplegia gradually improved which correlated to improvement in serum potassium levels. By day 3 of hospitalization, the quadriplegia had resolved. Serum creatinine, hypokalemia, and other electrolyte imbalance improved. On discharge, renal function, potassium, and bicarbonate were normal. Subsequently, he was started on pembrolizumab, another anti-PD-1 antibody, which the patient has tolerated well.

## 3. Discussion

As the use of immunotherapy in melanoma and other malignancies increases, the potential of adverse events (AEs) also increases. Safety reports for anti-PD-1's are available for > 2,000 patients participating in ongoing studies. Some side effects may be caused by an inflammatory mechanism [10]. Manufacturer information for nivolumab suggests that patients receiving nivolumab may develop immune-mediated nephritis and renal dysfunction (1.2% (23/1994) of patients). The median time to onset was 4.6 months (range: 23 days to 12.3 months) [11]. Immune-mediated nephritis and renal dysfunction led to permanent discontinuation of the medication in 0.3% and withholding of it in 0.8% of patients. All patients received high-dose corticosteroids (at least 40 mg prednisone equivalents per day) for a median duration of 21 days (range: 1 day to 15.4 months). Complete resolution occurred in 48% of patients [11]. Patients with immune-mediated nephritis present with some combination of the following laboratory findings: increased plasma creatinine, eosinophilia/eosinophiluria, characteristic urine sediment, and variable degree of proteinuria and rarely with evidence of tubulointerstitial damage such as Fanconi syndrome and renal tubular acidosis (RTA) [12]. A meta-analysis demonstrated that the use of immune checkpoint inhibitors was associated with an increased risk of immune-related renal toxicity [13]. As per common toxicity criteria (CTC), renal toxicity grade for urinary electrolyte wasting (e.g., RTA) is categorized from grade 0 to grade 4: grade 0 being none; grade 1 being asymptomatic, not requiring treatment; grade 2 being mild, reversible, and manageable with oral replacement; grade 3 being reversible but requiring IV replacement; and grade 4 being irreversible, requiring continued replacement. Grade 3 and 4 are considered as high-grade toxicity [14]. Three studies compared nivolumab with other chemotherapeutic agents in advanced melanoma. The incidence of all-grade renal toxicities in the nivolumab treatment arm ranged from 0.7 to 1.9% versus high-grade toxicities which ranged from 0 to 0.5% [13]. In our patient, based on the clinical presentation, the renal toxicity was grade 3. To our knowledge, this is the first case report of type 1 RTA associated with severe hypokalemia, caused by an immune checkpoint inhibitor. The patient had a basal metabolic profile studies prior to administering the medication, which was within normal range. Initial severe hypokalemia prompted further workup which led to identifying and characterizing his dysfunction. The differential diagnosis for RTA in a patient with no prior history of kidney disease includes medications, autoimmune disease particularly Sjögren syndrome or RA, kidney transplantation, nephrocalcinosis, medullary sponge kidney, chronic obstructive uropathy, cirrhosis, and sickle cell anemia [15]. A complete workup done ruled out most of these causes. Timing of his symptoms and the associated use of nivolumab, in the absence of any other precipitating factors (other medications and infection), pointed to immunotherapy being the most likely cause. The mainstay of immune-related adverse event (irAE) management is immune suppression, including high-dose steroids, immunosuppressants, and potentially TNF-α inhibitors [16]. Several specific algorithms are being developed for the management of the anti-PD-1 AEs. For most grade 1 toxicities, treatment can be continued with careful monitoring and supportive measures. Grade 2 toxicities usually resolve by withholding the drug and with administration of steroids. For grade 3 and 4 toxicities, discontinuation of the offending agent, use of high-dose corticosteroids, and a renal consultation and consideration for imaging or renal biopsy as appropriate are indicated [10]. Restarting of anti-PD-1 antibodies depends on the severity of the AEs. For majority of grade 1 and 2 toxicities, the drug can be safely but carefully restarted. In patients with grade 3 toxicity, retreatment requires close evaluation of the risks. Patients with grade 4 toxicity should not be rechallenged [3].

## 4. Conclusion

In summary, our case highlights a rare yet serious irAE of anti-PD-1 antibodies. Immune checkpoint inhibitors are an important advancement in the treatment of various cancers; however, this comes at the expense of a unique pattern of irAEs. Renal toxicity is rare but could be an important cause of morbidity in these patients [10]. Close monitoring of patients and a high degree of clinical suspicion is recommended when treating patients with immune checkpoint inhibitors. Early intervention with either careful monitoring of the patient or discontinuing the offending agent is the key to the management of these complications.
